# Refractory convulsive syncope in pregnancy: a rare presentation of Takayasu's arteritis - a case report and literature review

**DOI:** 10.4314/ahs.v21i2.46

**Published:** 2021-06

**Authors:** Gasthony Alobo, Violah Nahurira, Venice Omona, Pontius Bayo, Sam Olum

**Affiliations:** 1 Department of Obstetrics and Gynecology, Lacor Hospital, Gulu, Uganda; 2 Department of Obstetrics and Gynecology, Lira University, Lira Uganda; 3 Department of Pediatrics and Neurology, Lacor Hospital, Gulu, Uganda; 4 Department of Internal Medicine, Gulu University, Gulu, Uganda

**Keywords:** Takayasu's Arteritis in pregnancy, convulsive syncope, case report

## Abstract

**Background:**

Neurological manifestation of Takayasu's Arteritis (TA) in pregnancy presenting as convulsive syncope is extremely rare, and poses a serious diagnostic dilemma due to other vast causes of fits in pregnancy.

**Objective:**

We aimed to present and shed more light on a case of TA with convulsive syncope in pregnancy refractory to anticonvulsants for seven weeks, and review the literature on the management of TA in pregnancy.

**Case presentation:**

A gravida 4 para 3+0 at 28 weeks of amenorrhea presented with repeated episodes of the sudden loss of consciousness, followed by a fall and jerking of the limbs. These were refractory to anticonvulsants that she had used for seven weeks. Physical examination revealed undetectable pulse and blood pressure (BP) in the upper limbs but elevated BP in the lower limbs. Further investigations confirmed TA and she improved on steroids and antihypertensives.

**Conclusion:**

This case typically describes the unexpected presentation of TA with convulsive syncope. It calls for meticulous clinical assessment of epileptic seizures in pregnancy to avoid a late diagnosis of TA and its potential poor outcomes.

## Background

Takayasu's Arteritis (TA) is a rare chronic vasculitis of the large vessels, mainly the aorta and its branches 1. TA was named after Japanese Ophthalmologist Mikito Takayasu who together with his colleagues first described and reported a case of the disease in 1905^2^. The vascular lesions are typically characterized by stenosis, occlusion, dilation, or aneurysm of the large arteries 3. It predominantly affects females within the reproductive age 4, 5. Patients with TA may be asymptomatic or present with signs and symptoms that result from lesions such as dizziness, claudication, reduced or absent peripheral pulses, hypertension and/or blood pressure discrepancies between the arms, or the arms and lower limbs 6–9.

Although pregnancy does not have much effect on the progression of TA, the disease usually becomes apparent in the second and third trimesters. Pregnancy is also associated with alteration in cardiac and hematologic functions, therefore cardiovascular injury and thromboembolic events are feared complications of TA 10. There is a likelihood that such complications can result in poor maternal and fetal outcomes. Early diagnosis and prompt management of TA in pregnancy are therefore very important and this requires a multi-disciplinary approach involving obstetricians, rheumatologists, and cardiologists. However, in settings where TA is extremely rare, the diagnosis can be easily missed if the presentation is also unusual. Here we describe a 28-year old pregnant woman who was referred to our high-risk antenatal clinic (ANC) at Lacor Hospital in northern Uganda as a case of refractory convulsions in pregnancy.

## Case presentation

A 28-year-old woman of African origin, gravida 4 para 3+0 at 28 weeks of amenorrhea was referred to Lacor Hospital from a nearby district hospital due to repeated episodes of loss of consciousness, collapse, and tonic jerking of the limbs. These symptoms had occurred over seven weeks and each would last for a few seconds only. She noted that these episodes were common on standing up just before making any steps. Her care-takers also reported associated incontinece of urine occasionally. She did not have any known chronic illness or contact with Tuberculosis. The past three pregnancies were unremarkable as she did not attend antenatal care clinics and delivered at home with the traditional birth attendant (TBA) due to socioeconomic reasons. During this fourth pregnancy, she had booked for ANC at the district hospital with support from a humanitarian organization. The booking blood pressure at 11 weeks was 125/85 mmHg (taken from the upper limbs). She was started on anticonvulsants; phenytoin 200 mg two times a day for 4 weeks then switched to sodium valproate 800 mg per day for 3 weeks but the symptoms remained unchanged.

**Fig 1:**
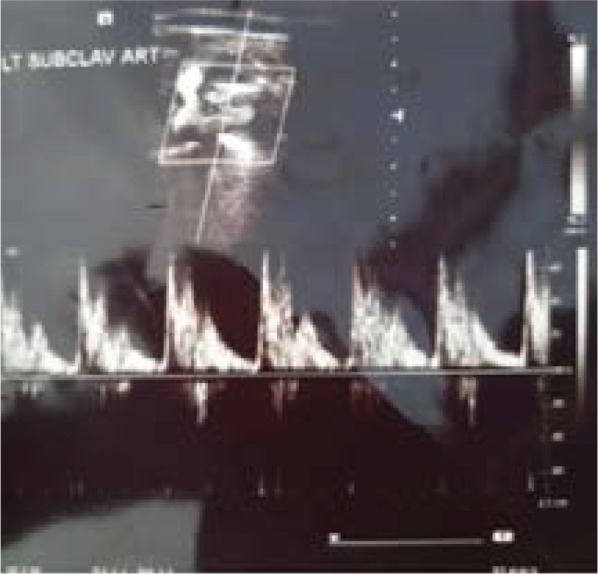
Left subclavian artery Doppler of the patient

**Fig 2:**
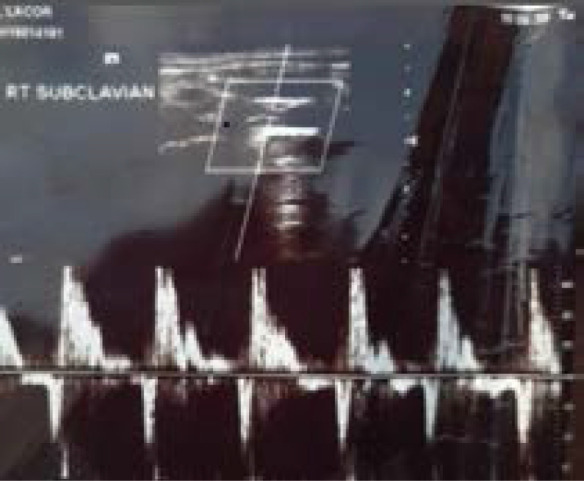
Right subclavian artery Doppler of the patient

**Fig 3:**
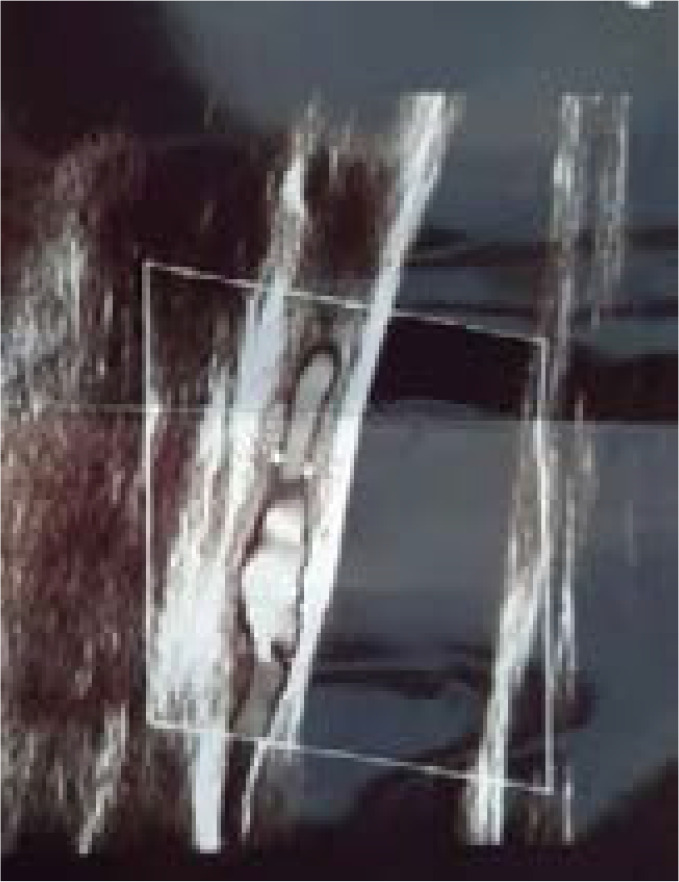
Doppler ultrasound of the left vertebral artery showing reverse flow (subclavian steal syndrome)

On arrival at Lacor Hospital, the carotid, brachial, and radial pulses were undetectable and the BP in the upper limbs was unrecordable. The BP from the ankle was 160/100 and pulse rate of 84 beats/minute. Per Abdomen examination revealed a symphysio-fundal height corresponding to 28 weeks with a fetal heart rate of 140 beats/minute that was regular. The other physical examinations were unremarkable. Investigations done include; echocardiogram which revealed normal cardiac chambers and valves. The ejection fraction was 58.57%. A Doppler scan of the aortic arch, descending aorta to iliac artery and renal artery, and electroencephalography findings were normal. There was thickening of the common carotid artery (CCA) and subclavian artery (SCA) with a 50–60% stenosis on Doppler (Figures 1 and 2). Also, a reverse flow pattern was noted within the vertebral artery referred to as subclavian steal syndrome (Figure 3). The erythrocyte sedimentation rate (ESR) was 40 mm/1st hour. Antinuclear antibody (ANA) and C-reactive protein (CRP) tests were not avalable at our facility. There was no proteinuria on spot urine dipstick. An obstetric ultrasound scan revealed a single live intrauterine fetus with a heart rate of 138/minute, a normal amniotic fluid index (AFI 11 CM), and normal umbilical artery Doppler velocimetry.

After reviewing the findings, we made a diagnosis of Takayasu's Arteritis with convulsive syncope. The patient was started on nifedipine 20 mg twice daily, with prednisolone 30 mg once a day; anticonvulsants were stopped. She was observed for one week on this treatment, the symptoms subsided and the ankle BP came down to 145/90 mmHg. The patient was discharged, and the dose of prednisolone tapered down to 10 mg daily over 4 weeks. She had a normal vaginal delivery at term to a male baby weighing 4.0kg. During the post-natal review, her BP was recorded as 115/75 mmHg and pulse of 80/minute in the upper limbs and she had no symptoms.

## Discussion

Takayasu's arteritis is a rare chronic granulomatous vasculitis of mainly the aorta and its branches. This can result in stenosis of the aortic arch, carotid, subclavian, iliac, or the abdominal aorta and renal arteries^11^. The patient presented here had involvement of the branches of the aortic arch only. TA is distributed worldwide but with the highest incidence in Japan, the eastern part of Asia, and India 12. The etiology of TA is largely unknown. However, autoimmunity, human leukocyte antigen (HLA – BW52, B40, DR2, DR4, DR7, DW3, DW12), and sex hormones have been postulated as plausible associations 13. Due to limited diagnostic modalities, we could not evaluate for the presence of these factors in the case described. Although chronic infectious diseases like tuberculosis (TB) have also been implicated as a possible trigger of TA 14, our patient did not give a history of any chronic illness likely to be TB. Pregnancy per se does not affect the progression of TA, but there are known maternal and fetal complications of TA. These complications result from narrowing of the aorta, aortic arch and its branches, and occasionally the pulmonary artery. The fetal complications include abortions, intrauterine growth restriction (IUGR), fetal death (FD), and rarely, placental abruption 15. Fetal complications are closely related to the reduced blood supply to the uterus from the narrowed abdominal aorta or iliac artery, and maternal cardiac insufficiency 16. This case did not have fetal complications and carried the pregnancy up to term; possibly due to non-stenosis of the aortic arch, abdominal aorta, and iliac vein. Nevertheless, it is recommended that all patients with TA in pregnancy should have a routine assessment of blood flow within the uterine and umbilical arteries 17.

Maternal complications include hypertension, aortic regurgitation, and aneurysm 16, 18. These complications are common in the second and third trimesters and can result in serious maternal morbidity and mortality^19^. Hypertension that results from TA is aproteinuric” unless there is superimposed pre-eclampsia; like in the case described. The increase in BP results from abnormal function of the baroreceptors at the carotid and aortic arch, and inelastic stenosis of the arteries 20. With the exception of hypertension, we did not observe any serious maternal complications in our case. But in other case reports the following have been documented; congestive cardiac failure, stroke, pre-eclampsia, eclampsia, and sudden death 21.

Making a diagnosis of TA in pregnancy is usually challenging in settings where the disease is rare because of the low index of suspicion as was the case with our patient. Diagnosis is based on clinical presentation, imaging to demonstrate thickening with stenosis of the aorta and its branches, and laboratory markers of acute-phase reactants. The clinical manifestation in pregnancy may range from asymptomatic to alteration in blood pressures and pulses, myalgia, claudication, and fainting 8. This patient presented with loss of consciousness, collapse, and tonic convulsions. The differential diagnosis of this neurological manifestation in pregnancy is quite broad and can be misleading. In our case, this led to a misdiagnosis of epilepsy and unnecessarily prolonged use of anticonvulsants in pregnancy. It is recommended that BP in patients with TA should be measured in both upper and lower limbs to detect any discrepancies, in addition to checking for bruits. In a study by Comarmond C. et al, it was found that new-onset or worsening arterial hypertension was evident in 27% of pregnant women with TA, but they noted that this could be an underestimate because in a few patients there may be stenosis of the vessels supplying all the four extremities, giving a misleadingly low blood pressure recording 16.

The computed tomography (CT) and magnetic resonance imaging (MRI) angiography can detect vascular changes of TA at the earliest stage 22. However, the only readily available imaging modality in our setting was ultrasonography. Nevertheless, in resource-limited settings, Doppler ultrasonography can be useful in elucidating vascular stenosis from the thickening of the arterial walls. In some studies, the FDG-PET scan was found to have a sensitivity 70.1% and specificity of 77.2%^23^; but this is still limited in resource-constrained settings. Laboratory findings include raised ESR and CRP, positive ANA, and rheumatoid factor (RF) 24. Our patient had a raised ESR of 40 mm/1st hours. It should be noted that during pregnancy there is also increased ESR due to the higher fibrinogen levels and CRP with unknown mechanisms. Therefore, the acute phase response is not mostly helpful to exclude inflammatory ischemic symptoms from physiological changes. We were unable to perform a vessel biopsy in our case, but vascular histology may be important to distinguish TA from other forms of arteritis when findings are not clear-cut 25. We relied on clinical features and results from imaging to make a diagnosis of TA in our case after meeting the following criteria: age less than 40 years at presentation, reduced or absent pulses in the upper limbs, evidence of stenosis or occlusion of the major branches of the aorta, and systolic BP discrepancy in the limbs more than 10 mm Hg 26.

The medications used in our case were: nifedipine 40 mg/day which was adequate to control hypertension; and prednisolone 30 mg/day. The following medicines can also be used to control hypertension; alpha-methyldopa which is Class A; propranolol is safe up to the dose of 60 mg/day, above this dose it can cause IUGR and preterm birth (Class B); nifedipine dose higher than 60 mg/day is considered Class B as well 6. Although the current European League Against Rheumatism (EULAR) proposes a higher induction therapy for the treatment of vasculitis with an initial 1 mg/kg oral prednisolone (maximum 60 mg/day) for one month with subsequent tapering to 10–15 mg/day over some time 27, this dose may have adverse effects in pregnancy 10. There is evidence of good results with doses under 30 mg/day in active TA during pregnancy^11^. Patients who are resistant to steroids can benefit from immunomodulatory agents like infliximab, tocilizumab, leflunomide, and mycophenolate mofetil 28, 29. However, their safety in pregnancy is not well established and it is advisable to avoid using them unless the benefits outweigh the risks 28,29.

Our patient had spontaneous vertex delivery. There is evidence to favor vaginal delivery, either spontaneous or assisted, in hemodynamically stable patients. Cesarean delivery is recommended in those who are having decompensated cardiac function or another obstetric indication 18. Anesthesia is considered safe in patients with TA, and epidural anesthesia is preferred^30^.

Overall, neurological manifestations of TA are rare; even in the general population. Ischemia due to vascular stenosis initially presents with headache, dizziness, visual disturbance, transient ischemic attack, stroke, and very rarely convulsive syncope 18, 19. Convulsive syncope in pregnancy has not been reported; this could be the first case report. Our database searches with the following MeSH subheadings and Boolean operator (AND): “syncope” AND “convulsion” AND “Takayasu's arteritis” AND “pregnancy” yielded no results.

## Conclusion

TA in pregnancy presenting with convulsive syncope can pose a serious diagnostic dilemma. This may lead to misdiagnosis and unnecessary use of anticonvulsants. The unusual presentation that we have described here will help to shed more light on how TA can be differentiated from other causes of convulsions in pregnancy.

## Abbreviations

ANA: Anti-nuclear antibody; BP: Blood pressure; CCA: Common carotid artery; CRP: C-reactive protein; ESR: Erythrocyte sedimentation rate; EULAR: European League Against Rheumatism; IUGR: Intrauterine growth restriction; SCA: Subclavian artery; TA: Takayasu's arteritis; TBA: Traditional birth attendant.

## Declarations

### Availability of data and materials

All data generated or analyzed during this study are included in this published article.
